# COMMUNITY PREVALENCE OF HEARING LOSS AMONG OLDER ADULTA IN MA, RI, CT, AND NH: COMPARING MEASURES

**DOI:** 10.1093/geroni/igae098.3331

**Published:** 2024-12-31

**Authors:** Eric Frimpong, Alexa Fleet, Constanza Tamara Matta Gallero, Taylor Jansen, Elizabeth Dugan

**Affiliations:** University of Massachusetts Boston, Boston, Massachusetts, United States; University of Massachusetts Boston-- Gerontology, Boston, Massachusetts, United States; University of Massachusetts Boston, Boston, Massachusetts, United States; University of Massachusetts Boston, Boston, Massachusetts, United States; University of Massachusetts Boston, Boston, Massachusetts, United States

## Abstract

**Background:**

Hearing impairment and hearing loss may negatively impact social engagement and social relationships. Understanding community rates of hearing loss would identify disparities and may inform service delivery and potentially improve quality of life for adults with hearing loss. The aim of this study is to describe community prevalence of hearing loss and to compare two measurement approaches: self-report and clinically derived from Medicare claims.

**Methods:**

Data were from the Healthy Aging Data Report (healthyagingdatareports.org) for the New England states (MA, NH, CT, and RI) with indicators calculated from the American Community Surveys (MA: 2012-2016; RI:2016-2018; CT: 2014-2018 and NH: 2012-2016), and the Medicare Master Beneficiary Summary Files (MA: 2014-2015; RI: 2016-2017; CT: 2016-2017 and NH: 2014-2015).

**Results:**

rates derived from CMS clinical diagnosis were higher (MA: 16.1%; RI: 16.4%; CT: 15.7%, and NH: 14.4%) compared to the self-reported ACS rates (MA: 14.2%; RI: 13.9%: CT: 12.4% and NH: 15.0%). The highest community prevalence rate of self-reported hearing loss (36.36%) was in Monroe MA. For clinically diagnosed hearing loss, Hanover NH (home to Dartmouth College and health system) reported the highest rate (26.12%). Having excellent access to health care is associated with higher community rates of clinically diagnosed hearing loss. Results showed that the community prevalence of hearing impairment was variable by community. The potential impact of hearing loss on social, emotional, and physical well-being indicates a need for interventions to diagnose and treat it.

